# Effects of ZrO_2_ Nano-Particles’ Incorporation into SnAgCu Solder Alloys: An Experimental and Theoretical Study

**DOI:** 10.3390/nano14201636

**Published:** 2024-10-12

**Authors:** Agata Skwarek, Halim Choi, Tamás Hurtony, Jaeduk Byun, Ahmad Azmin Mohamad, David Bušek, Karel Dušek, Balázs Illés

**Affiliations:** 1 Łukasiewicz Research Network, Institute of Microelectronics and Photonics, 30-701 Kraków, Poland; agata.skwarek@imif.lukasiewicz.gov.pl; 2 Department of Electronics Technology, Faculty of Electrical Engineering and Informatics, Budapest University of Technology and Economics, 1111 Budapest, Hungary; inertia9192@gmail.com (H.C.); hurtony.tamas@vik.bme.hu (T.H.); 3 Department of Physics, Dankook University, Cheonan 31116, Republic of Korea; bjdpjq@dankook.ac.kr; 4 Energy Materials Research Group (EMRG), School of Materials and Mineral Resources Engineering, Universiti Sains Malaysia, Nibong Tebal 14300, Malaysia; aam@usm.my; 5 Department of Electrotechnology, Faculty of Electrical Engineering, Czech Technical University in Prague, 16627 Prague, Czech Republic; busekd1@fel.cvut.cz (D.B.); dusekk1@fel.cvut.cz (K.D.)

**Keywords:** nano-particles, ZrO_2_, SAC0307, soldering, Sn whisker, corrosion

## Abstract

This study investigates the mechanism and effects of incorporating different ZrO_2_ nano-particles into SAC0307 solder alloys. ZrO_2_ nano-powder and nano-fibers in 0.25–0.5 wt% were added to the SAC0307 alloy to prepare composite solder joints by surface mount technology. The solder joints were shear tested before and after a 4000 h long 85 °C/85% RH corrosive reliability test. The incorporation of ZrO_2_ nano-particles enhanced the initial shear force of the solder joint, but they decreased the corrosion resistance in the case of 0.5 wt%. SEM, EDS, and FIB analysis revealed intensive growth of SnO_2_ on the solder joint surfaces, leading to the formation of Sn whiskers. Density functional theory (DFT) simulations showed that, despite Sn being able to bond to the surface of ZrO_2_, the binding energy was weak, and the whole system was therefore unstable. It was also found that ZrO_2_ nano-particles refined the microstructure of the solder joints. Decreased β-Sn grain size and more dispersed intermetallic compounds were observed. The microstructural refinement caused mechanical improvement of the ZrO_2_ composite solder joints by dispersion strengthening but could also decrease their corrosion resistance. While ZrO_2_ nano-particles improved the solder joint mechanical properties, their use is recommended only in non-corrosive environments, such as microelectronics for space applications.

## 1. Introduction

The mechanical and electrical structure of microelectronic circuits is based on solder joints, which have made soldering an essential technology for microelectronics manufacturing for six decades. After the transition of the technology to lead-free in the early 2000s, research in soldering turned towards so-called composite soldering. Composite solder alloys are prepared when ceramic or rarely intermetallic (IMC) particles are incorporated into the solder joints. The particle size is usually in the nano-range and constitutes a 0.1–1 wt% of the solder matrix [1]. Three methods can be applied to prepare composite solders: the simplest and most commonly used is mixing nano-particles (NPs) into the solder paste; alternatively, NPs can be added to the solder powder (powder metallurgy) or during the alloying process [2].

Several ceramics, mostly TiO_2_, SiC, ZnO, and rarely Al_2_O_3_, ZrO_2_, Fe_2_O_3_, Si_3_Ni_4_, etc. were tested in SnCu, SnAg, SnAgCu (SAC), SnZn, and SnBi solder alloys [3,4,5,6]. Generally, ceramic NPs have favorable effects on composite solder joints’ mechanical properties [7,8], though they can cause slight shifts in the solidus–liquidus points (1–2 °C) of the composite alloys [9]. Mechanical strengthening is caused by the dispersion and incorporation of ceramic NPs into the Sn-matrix. The non-soluble NPs incorporate between the phases of the Sn-matrix (β-Sn and IMC grains). The particles, with nanometer-scale dimensions, typically refine the grain structure of both Sn and IMCs through heterogeneous nucleation during the solder alloy’s solidification [10,11,12].

Incorporating ZrO_2_ into lead-free solder alloys was never as popular as incorporatingTiO_2_ or ZnO, but it has had an almost two-decade history. Shen et al. [13] prepared Sn3.5Ag-ZrO_2_ solder by mechanically stirring ZrO_2_ NPs (2 wt%) into the molten solder. They observed the significant refinement of the Ag_3_Sn IMCs due to the intensive adsorption effect of ZrO_2_ NPs, which increased Vicker’s microhardness of the composite alloy. Later, they successfully applied 1 wt% ZrO_2_ NPs to Sn-9Zn solder alloy [14]. It was found that ZrO_2_ NPs increased the shear strength and reliability of solder balls made from Sn-9Zn-1ZrO_2_ after multiple reflow cycles. ZrO_2_ NPs were embedded in the solder matrix to block dislocation motions by pinning the grain boundaries [14].

Gain et al. [15,16] were the first to incorporate 1–3 wt% of ZrO_2_ NPs into the classical SAC305 alloy. They investigated the microstructure and mechanical parameters of the solder joints after multiple reflow cycles. They observed considerable refinement of Ag_3_Sn and Cu_6_Sn_5_ IMCs, and β-Sn grains with increased hardness of the composite alloy from 15.0 Hv and 17.1 Hv [15]. Furthermore, they found a shear strength increase in the prepared composite solder joints caused by the second-phase dispersion strengthening mechanism. The fracture surface of the composite solder joints showed ductile failure characteristics with rough dimpled surfaces, in contrast to SAC305 which displayed a brittle fracture with a smooth surface [16]. Later, they conducted high temperature/mechanical damping tests, where the composite solder joints showed a lower damping capacity than pure SAC305 since the ZrO_2_ NPs (1 wt%) hindered dislocation motion and grain boundary sliding [17].

Hu and Chen [18] observed ductile failure with large dimples and plastic deformation during fracturing in the presence of ZrO_2_. They also proved that the usually observed suppression of the IMC layer growth is caused by the diffusion-blocking effect of the incorporated ZrO_2_ NPs on the Cu atoms at the solder joint interfaces. Sharma et al. [19] investigated the electromigration (EM) behavior of different composite SAC solder alloys, and they observed that, for SAC-0.5ZrO_2_ solder joints, the aging by current loading did not form voids and cracks in the composite joints as it did in SAC alone. Yakmovych et al. [20] investigated the microstructure and mechanical properties of SAC305-(SiO_2_/TiO_2_/ZrO_2_) solder joints where 0.5 and 1 wt% of ceramic NPs were added. They obtained similar positive results from the NPs, as discussed above; furthermore, they highlighted that, with higher ZrO_2_ weight fraction additions, the NPs were prone to agglomerate in the solder bulk.

Rajendran et al. [21] investigated the reliability of SAC305-0.2ZrO_2_ solder joints by isothermal aging. They found that the ZrO_2_ suppressed the growth rate of the Cu_6_Sn_5_ IMC layer, not only in the liquid state during the soldering but later in the solid state as well during the aging, which improved the reliability of the composite solders. Wodak et al. [22] got similar results during isothermal aging of SAC305 composite solder joints containing 0.2 or 0.5 wt% ZrO_2_ NPs, and they highlighted the risk of NPs’ agglomeration in the case of 1 wt%. Hou et al. [23] conducted surface modification of ZrO_2_ NPs with NiO NPs by ball-milling pyrolysis methods, and added different weight fractions of NiO-ZrO_2_ NPs (0.05–0.5 wt%) into SAC105 solder paste. They achieved optimal mechanical properties and microstructural stability during isothermal aging with a 0.3 wt% addition of ZrO_2_.

Mousa et al. [24] added 0.5 wt% of ZrO_2_ or TiO_2_ NPs into Sn–Zn–Cu–Ni (SZCN) solder alloy by powder metallurgy. They found that the SZCN-ZrO_2_ composite solder had the greatest hardness and stress exponent values due to the suppressed growth of β-Sn grains and the pile-up of dislocations. Mohamed et al. [25] investigated the impact of a minor addition of Ni and ZrO_2_ NPs to eutectic Sn-9wt%Zn (SZ) prepared by a vacuum melting technique. The third element in the solder bulk refined the β-Sn grain structure and enhanced the mechanical properties during the tensile test by 20%. ZrO_2_ NPs also had a positive effect on low-temperature solder (LTS) alloys. Nitta et al. [26] reinforced Sn–52In solder alloy with NiO-coated ZrO_2_ NPs in 0–0.5 wt%. Their experimental results showed that the addition of NPs increased the tensile strength by 35.6% (0.4 wt% NPs), which was attributed to grain refinement and dispersion strengthening. Singh et al. [27] investigated the influence of Mo or ZrO_2_ NPs in 0.3 wt% on the interfacial properties and shear strength of a Sn58Bi solder joint. They observed an increase in the average maximum load and shear stress of the Sn58Bi + ZrO_2_/Cu solder joints by 69%.

According to the former studies, ZrO_2_ NPs were typically used in 0.1–1 wt% in the composite solder joints; only some examples are available in the literature for lower or higher weight fractions. Mechanical improvements of the composite solder joints were mostly observed in the case of 0.2–0.5 wt%. The connection between the microstructural refinement and the mechanical improvement of the composite solder joints can be explained by the dispersion strengthening theory [28,29]. Nearly uniformly distributed fine particles, like ZrO_2_ NPs and the refined dispersed IMCs, can improve the mechanical properties of the Sn-matrix by the pinning of grain boundaries, thus impeding the sliding of the grain boundaries and by increasing the dislocation density in the matrix and obstacles, thus restricting the motion of dislocation [28,29].

Recent studies have shown that ceramic NPs can have varying effects on the reliability of composite solders. TiO_2_ and ZnO increased the corrosion resistance and decreased the Sn whisker formation from composite SAC0307 solder alloys [30], however, SiC addition had the totally opposite effects [31]. The ZrO_2_ NPs are promising candidates for soldering technologies, but their effect on the corrosion reliability of the composite solder joints remains uncertain since the previous studies were mostly limited to isothermal aging.

## 2. Materials and Methods

A low Ag content solder alloy, the Sn99Ag0.3Cu0.7 (SAC0307, Stannol Industries), in solder paste format, was used during the investigations. ZrO_2_ nano-powder (np) and nano-fibers (nf) at concentrations of 0.25 wt% and 0.5 wt% were used to prepare composite solders. Such low weight fractions were used to avoid agglomeration of the particles in the Sn-matrix [20] and to reach good microstructural stability of the solder joints [23]. The ZrO_2_ (by Sigma Aldrich, Burlington, MA, USA) had the following dimensions: nano-powder (np) with primary particle sizes of less than 100 nm, and nano-fibers (nf) with diameters of 200–800 nm ± 100 nm and lengths of 2–10 μm (polycrystalline). The homogeneous distribution of the NPs in the solder paste was achieved by a YX solder paste mixer (400 rpm/15 min). Five types of solder were investigated: SAC0307-0.25ZrO_2_(np), SAC0307-0.5ZrO_2_(np), SAC0307-0.25ZrO_2_(nf), SAC0307-0.5ZrO_2_(nf), and a reference SAC0307.

0603-SMD chip resistors were soldered with the above-listed solder pastes on FR4-printed circuit boards (PCBs). The test boards had solder pads made of copper foil coated with an im-Ag surface finish. Standard surface mounting technology (SMT) was applied: solder deposition by screen printing, component placement by a manual pick-and-place machine, and reflow soldering by an infrared batch oven. A linear thermal profile with the following setting was used: preheating (150 °C/0–120 s), soak (150–190 °C/120–240 s), and ramp-up (255 °C/240–320 s). The solder joints were subjected to an 85 °C/85%RH temperature-humidity (TH) test for 4000 h to assess their corrosion reliability. The shear force of the solder joints was measured using a DAGE BT2400 shear force tester after sample preparation (0 h), then after 2000 h of TH test, and finally after 4000 h of TH test. Twenty resistors from each sample type were measured at each stage.

The solder joints were observed after each 1000 h of the test by a Thermo Scientific Quatro scanning electron microscope (SEM). Corrosion spots and corrosion-induced Sn whiskers [30,31] were the objects of the observation. Five chip components (10 solder joints) were examined from each solder type. The Sn whisker length was determined from their tip to the surface of the solder joints (according to JESD201 standard [32]). FIB cuts were prepared and analyzed on the samples by a Thermo Scientific Scios 2 focused ion beam (FIB) to study the microstructure of the solder joints. Elemental compositions were measured by Bruker energy dispersive X-ray spectroscopy (EDS). The solder joints were cross-sectioned at the end of the 85 °C/85% RH TH test to study the corrosion level within. The corrosion level of the solder joints was quantitatively compared with the spatial corrosion depth [33] as a metric.

The potential interaction between Sn and ZrO_2_ was investigated using density functional theory (DFT) calculations and simulations. They were performed within the plane-wave/pseudopotential formalism as implemented in the Quantum ESPRESSO package using the Perdew–Burke–Ernzerhof (PBE) exchange-correlation functional.

## 3. Results

### 3.1. Shear Forces of the Solder Joints

**Figure 1:**
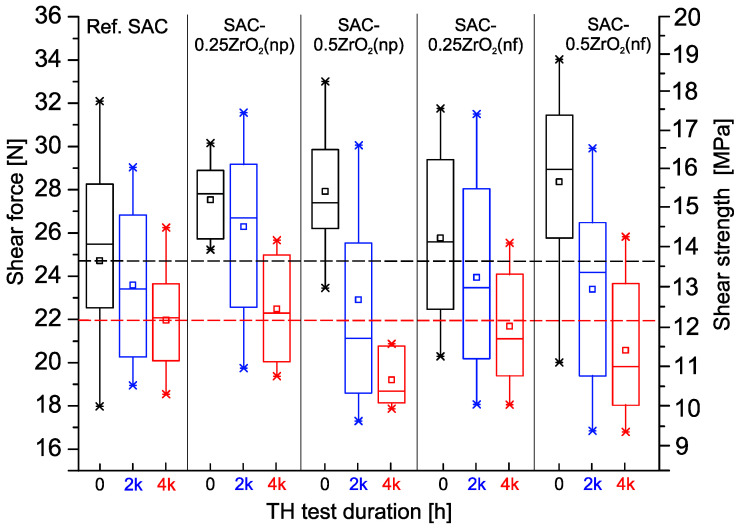
Statistics of the shear forces.

The shear force (strength) is one of the most important quality and reliability parameters during the life cycle of the solder joints. Figure 1 shows the statistical results of the shear forces and strengths obtained during the study in box plots. The following markings were applied in Figure 1 for easier evaluation of the results: the black dashed line represents the average of the reference SAC0307 at 0 h (24.9 N), and the red dashed line shows the same parameter after 4000 h (22 N) of the TH test.

The reference SAC0307 joints had 24.9 N average shear force with a relatively high deviation after their preparation (0 h). The ZrO_2_ composite solder joints showed higher averages between 25.9 N and 28.2 N. The ZrO_2_ nano-powder increased by 10.5% (0.25 wt%) and by 12.5% (0.5 wt%), while the ZrO_2_ nano-fibers increased by 4% (0.25 wt%) and by 13.2% (0.5 wt%). This meant a considerable quality improvement of the composite solder joints. Generally, the shear force increase was proportional to the weight fraction of the NPs. Furthermore, with the application of ZrO_2_ nano-powders, the composite solder joints were more stable (the deviation in the shear force was considerably lower) than the reference ones. However, composite solder joints containing nano-fibers exhibited higher deviations. The initial shear force of the 0603 SMDs is usually in the range of 20–30 N, depending on the applied solder alloy, soldering technology, and thermal profile [34,35,36]. The obtained results fit this trend.

The reference SAC0307 joints dropped to 23.6 N (−5%) average shear force with a still high deviation after 2000 h of the TH test. All ZrO_2_ composite joints experienced a reduction in shear force, though to varying degrees: SAC-0.25ZrO_2_(np) decreased to 25.9 N (−6%), SAC-0.5ZrO_2_(np) to 22.9 N (−18%), SAC-0.25ZrO_2_(nf) to 24 N (−7.5%), and SAC-0.5ZrO_2_(nf) to 23.5 N (−17%). The degradation of the composite solder joints with 0.25 wt% NPs was similar to the reference ones, while the higher 0.5 wt% NP content caused higher shear force degradation. The composite joints’ shear force deviations also increased, indicating a loss in stability. After 2000 h of TH test, only the SAC-0.25ZrO2(np) composite solder joints maintained higher shear force than the reference solder joints; the rest of the composite joints were at the same level as the reference.

During the second half of the test, the shear forces continuously decreased. The reference SAC0307 joints dropped to 21.9 N (further −7%), SAC-0.25ZrO_2_(np) to 22.5 N (−13%), SAC-0.5ZrO_2_(np) to 19.1 N (−17%), SAC-0.25ZrO_2_(nf) to 22.8 N (−5%), and SAC-0.5ZrO_2_(nf) to 20.7 N (−12.5%). The deviation of the shear force decreased in each case, which meant that during the 4000 h TH test, most of the solder joints degraded considerably. Generally, ZrO_2_ composite joints exhibited better mechanical properties immediately after preparation (0 h) compared to the reference SAC0307. Composite solder joints, including 0.25 wt% ZrO_2_ NPs, showed similar reliability to the reference ones. The reference SAC0307 joints lost 12% of their strength, while the composite ones (with 0.25 wt%) lost 12.5% (nf) and 19% (np) of their shear forces. Composite joints, including 0.5 wt% ZrO_2_ NPs, showed worse reliability than the reference ones. They lost 29% (nf) and 35% (np) of their shear forces. So, at the end of the 85 °C/85% RH TH test, the composite joints with 0.25 wt% ZrO_2_ NPs exhibited similar shear force statistics as the reference SAC0307; the composite joints with 0.5 wt% ZrO_2_ NPs exhibited worse statistics (Figure 1).

### 3.2. Surface Defects on the Solder Joints

**Figure 2:**
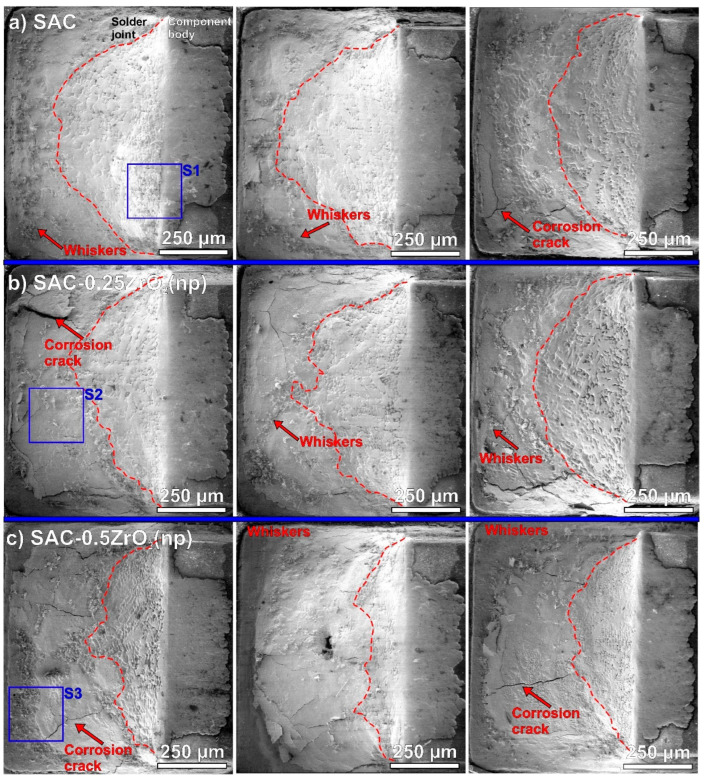
SEM micrograph of the surface of the joints after 4000 h TH test: (**a**) ref. SAC; (**b**) SAC-0.25ZrO_2_(np); (**c**) SAC-0.5ZrO_2_(np).

**Figure 3:**
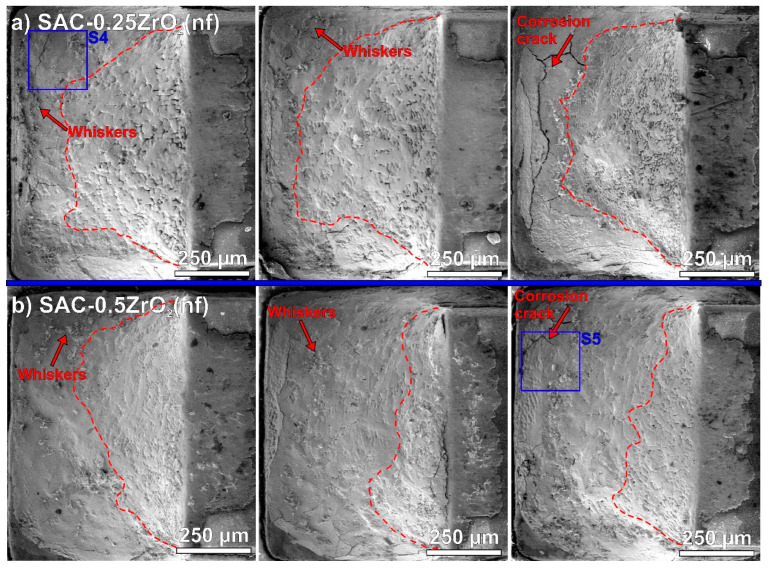
SEM micrograph of the surface of the solder joints after 4000 h TH test: (**a**) SAC-0.25ZrO_2_(nf); (**b**) SAC-0.5ZrO_2_(nf).

**Figure 4:**
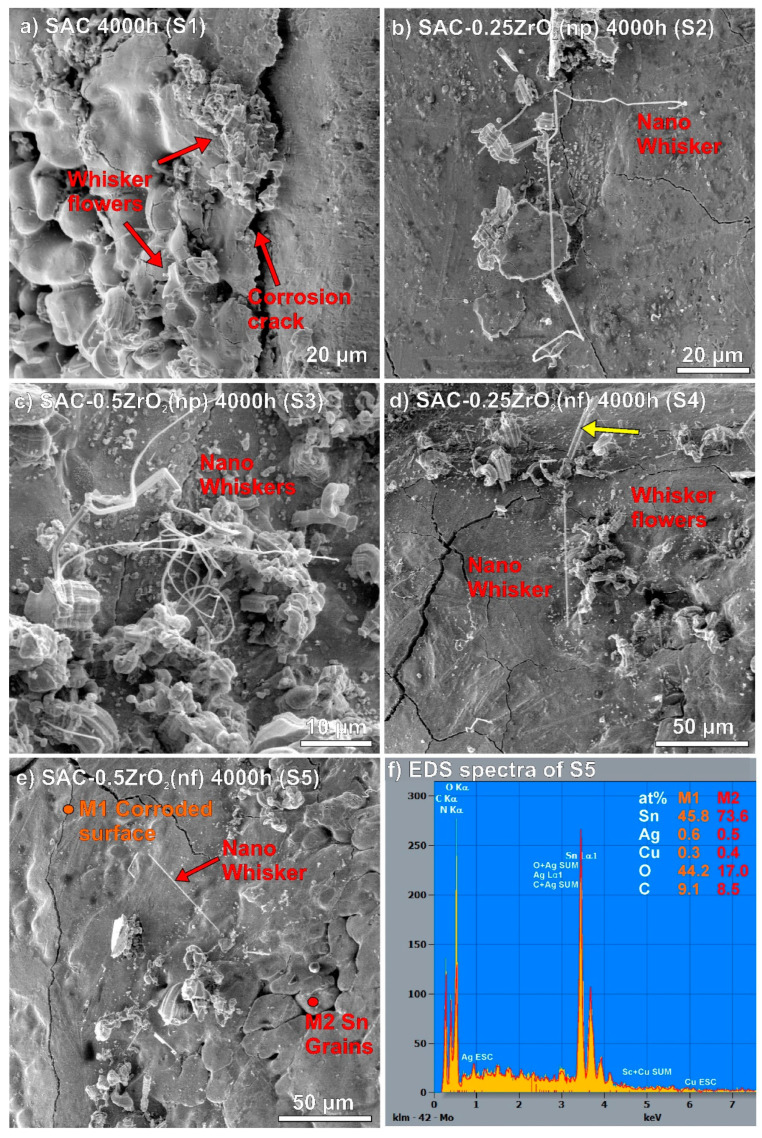
Sn whiskers on the solder joints: (**a**) reference SAC; (**b**) SAC-0.25ZrO_2_(np); (**c**) SAC-0.5ZrO_2_(np); (**d**) SAC-0.25ZrO_2_(nf); (**e**) SAC-0.5ZrO_2_(nf); (**f**) EDS spectra of S5.

Figure 2 and Figure 3 show SEM micrographs of the solder joints from the upper view after the 4000 h TH test, and three samples from each type. Figure 2 presents the reference SAC0307 and the SAC-ZrO_2_(np) samples. The signs of considerable corrosion were observable on all solder joints, but the corrosion levels differed. The non-corroded and corroded areas could be clearly differentiated by the surface morphologies. Sn grains were observable in a non-corroded area, while a corroded area showed a continuous and generally smoother layer (Figure 4e,f present a detailed analysis of this issue with EDS results). The red dashed lines mark approximated borders between the non-corroded and corroded areas. The lower meniscus was always more corroded than the upper parts of the solder joints since the condensed water accumulated there during the TH test. The SAC-0.5ZrO_2_(np) composite solder joints (Figure 2c) corroded more than the reference SAC0307 and SAC-0.25ZrO_2_(np) (Figure 2a,b). The corroded areas were larger, and longer corrosion cracks were typical on the surfaces of SAC-0.5ZrO_2_(np) samples compared to the rest of the samples. The considerable corrosion of the SAC-0.5ZrO_2_(np) composite solder joints could be a reason for their shear force decline during the TH test (Figure 1).

Numerous Sn whiskers were found in and around the corroded areas. Sn whiskers are dangerous surface effects that develop due to mechanical stress on high tin-content objects. Tin whiskers can easily reach lengths of hundreds of micrometers. Since they are electrically conductive, they pose a significant reliability risk for fine-pitch electronic components due to the potential for short circuit formation. In our experiment, the oxidation of Sn grains caused mechanical stress supporting whisker development. Tin has a higher density than its oxides (SnO and SnO_2_), therefore, the oxidation of an Sn layer causes a volume increase, which results in mechanical stress, as presented in [30,31]. There was no apparent difference in the whiskering between the reference SAC0307 and the SAC-ZrO_2_(np) samples.

Figure 3 presents SEM micrographs of the surface of SAC-ZrO_2_(nf) solder joints. These results were similar to the results achieved with the nano-powder (Figure 2). SAC-0.5ZrO_2_(nf) samples (Figure 3b) exhibited more severe corrosion compared to the reference SAC0307 (Figure 2a) and the SAC-0.25ZrO_2_(nf) composite joints (Figure 3a). Initial examination revealed fewer whiskers on the SAC-ZrO_2_(nf) samples compared to the reference samples.

Figure 4 presents some Sn whiskers in a higher magnification. The locations of the observed Sn whiskers were marked with S1–S5 blue rectangles in Figure 2 and Figure 3. Typical effects of corrosion-induced Sn whiskers are visible in Figure 4. The corrosion during the TH test resulted in numerous nodule-type whiskers and arranged them to flower-like shape (Figure 4a,d). The surface of the whiskers was quickly oxidized as well, which twisted their body and blocked their further length development. Furthermore, filament whiskers were also observed (Figure 4b–e). Interestingly, very thin, long, and occasionally twisted filament whiskers were also found, mostly on the surface of ZrO_2_ composite solder joints (Figure 4b–e). Their thickness was below 500 nm, so we refer to them as “nano-whiskers”. The thickness of the filament whiskers is usually above one micron. An example of an average filament-type whisker is shown by a yellow arrow in Figure 4d; it was approximately 6 µm in diameter. The growth of the nano-whiskers could be caused by the microstructural refinement of the composite solder joints [10]. The whisker thickness is usually correlated with the Sn grain size [37]. Figure 4e presents an example of the above-discussed difference between the corroded (M1) and non-corroded (M2) areas. The EDS results also showed considerable oxygen differences between the M1 and M2 areas (Figure 4f).

We counted the Sn whiskers and measured their length using self-developed automatic image-processing software [38]. Table 1 contains the obtained statistical parameters: whisker density/1 mm^2^, average lengths, and maximum detected lengths. The reference SAC0307 solder joint produced 396 pcs./mm^2^. The number of Sn whiskers for SAC-ZrO_2_(np) composite solder joints increased by approximately 10%. SAC-ZrO_2_(nf) composite solder joints produced almost the same number of Sn whiskers as the reference SAC0307. The correlation between the intensity of whiskering and the size of the corroded areas was weaker than initially expected (Figure 2 and Figure 3). It is interesting since corrosion was the main inducing factor for the whisker growth [30]. However, the ZrO_2_ composite solder joints produced slightly longer Sn whiskers than the reference SAC0307, but their number was similar. The maximum length of the whiskers was usually below 100 µm on all sample types. Only one whisker longer than 100 µm was found on a SAC-0.5ZrO_2_(np) composite solder joint. Overall, it can be concluded that the use of ZrO_2_ NPs did not increase the reliability risk of whisker growth in the composite solder joints.

### 3.3. Corrosion Depth in the Solder Joints

**Figure 5:**
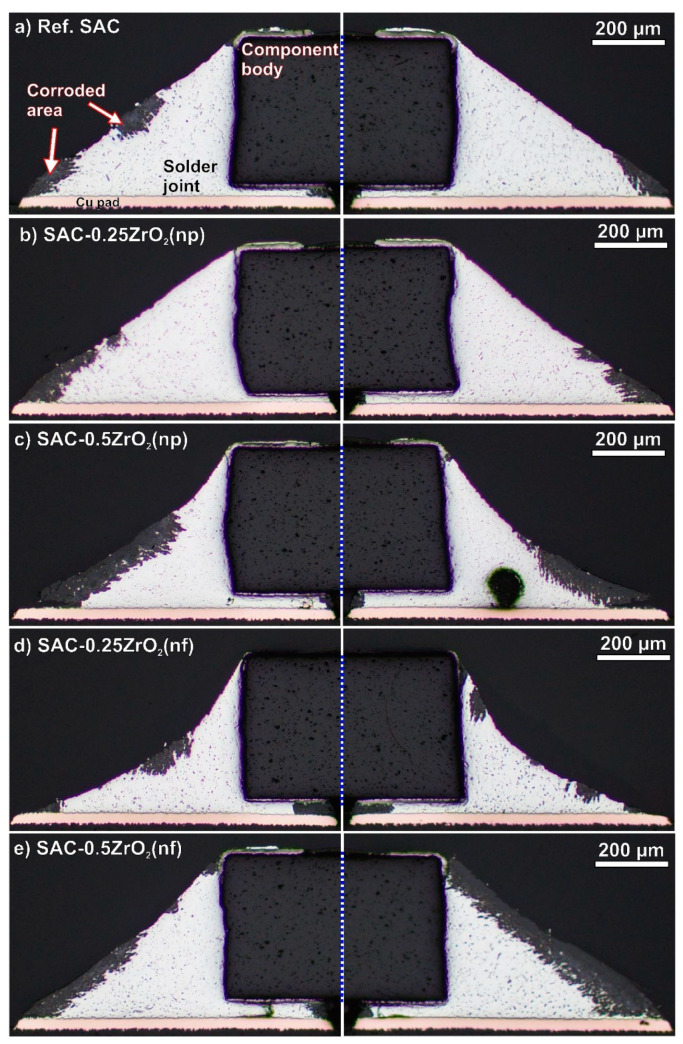
Cross-section of the solder joints after 4000 h TH test: (**a**) ref. SAC; (**b**) SAC-0.25ZrO_2_(np); (**c**) SAC-0.5ZrO_2_(np); (**d**) SAC-0.25ZrO_2_(nf); (**e**) SAC-0.5ZrO_2_(nf).

**Figure 6:**
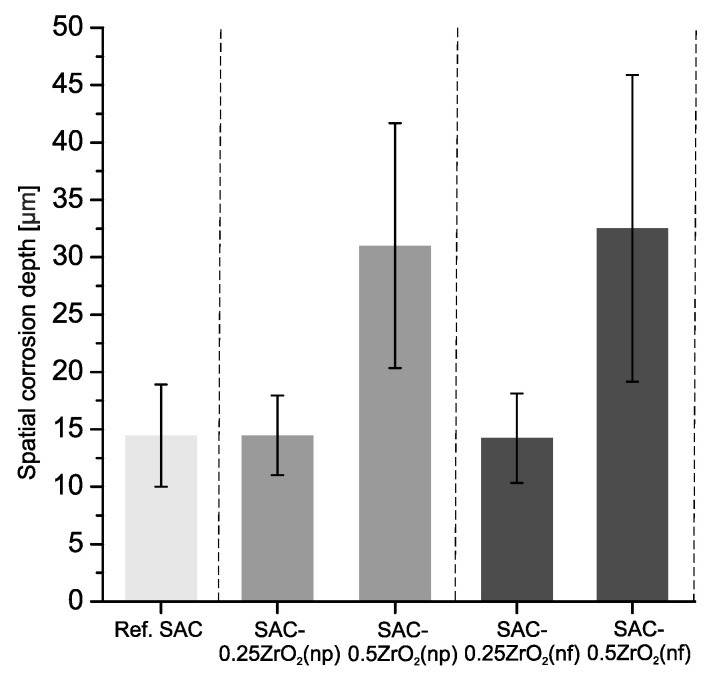
Spatial corrosion depth (d*_SC_*) in the solder joints.

The solder joints were cross-sectioned to assess their corrosion depths after the 4000 h TH test. Figure 5 presents 2-2 cross-sectioned solder joints of each type. The cross-sectional analysis confirmed our conclusions based on the surface evaluations shown in Figure 2, Figure 3 and Figure 4. All solder joints exhibited deep corrosion spots, though the extent varied. The reference SAC0307 solder joints (Figure 5a) and the composite joints with 0.25 wt% ZrO_2_ (Figure 5b,d) showed a similar extent of corrosion spots, while the composite ones with 0.5 wt% ZrO_2_ (Figure 5c,e) experienced significantly more severe corrosion damage. The level of corrosion in the different solder joints was quantitatively compared using spatial corrosion depth (d*_SC_*). This was calculated based on the average corrosion depth, weighted by the corroded length of the solder meniscus [33]. Figure 6 shows the d*_SC_* values in the different solder joints.

The spatial corrosion depth was much deeper in the composite solder joints with 0.5 wt% ZrO_2_ than in the rest of the samples. The SAC-0.5ZrO_2_(np/nf) solder joints exceeded 30 µm on average, while the rest of the samples were below 15 µm on average.

## 4. Discussion

### 4.1. Microstructural Differences of the Composite Solder Joints

**Figure 7:**
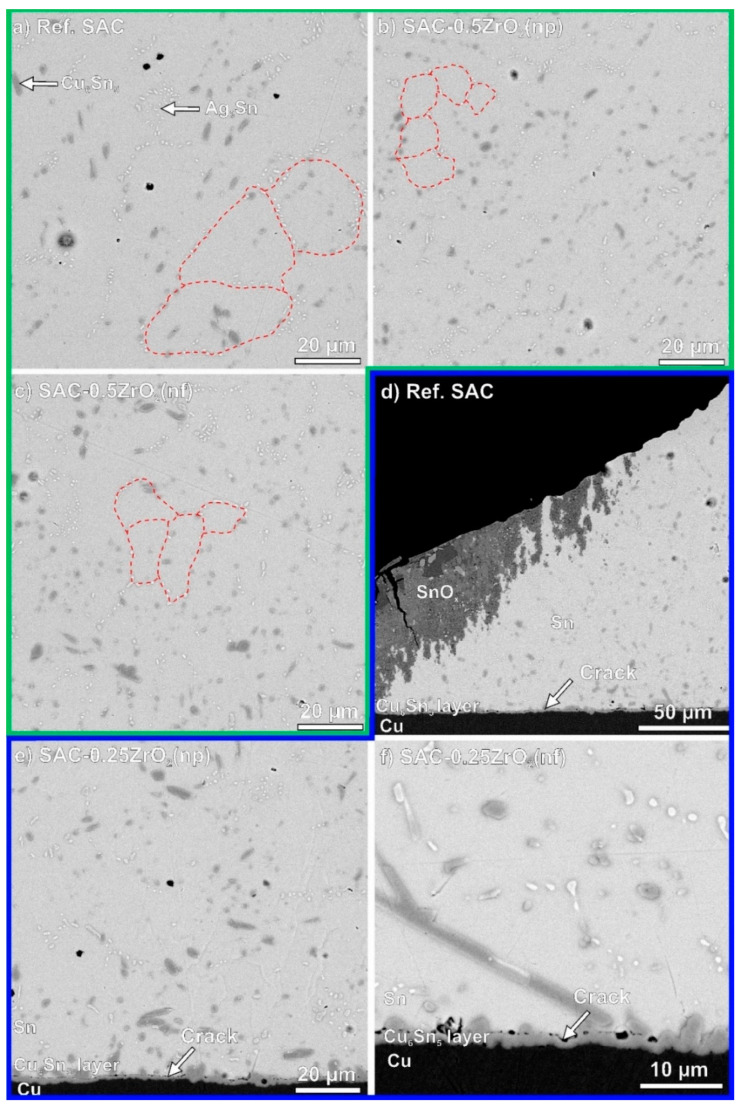
Microstructure of the solder joints: (**a**) Ref. SAC, 0 h TH test; (**b**) SAC-0.5ZrO_2_(np), 0 h TH test; (**c**) SAC-0.5ZrO_2_(nf), 0 h TH test; (**d**) ref. SAC, 4000 h TH test; (**e**) SAC-0.25ZrO_2_(np), 4000 h TH test; (**f**) SAC-0.25ZrO_2_(nf), 4000 h TH test.

The incorporation mechanisms of ZrO_2_ NPs need further investigation to explain their effects on the properties of composite solder joints. As discussed in the Section 1, the non-soluble ZrO_2_ NPs were dispersed and incorporated between the Sn and IMC grains. During the solidification of the solder alloy, the NPs enhanced grain growth through heterogeneous nucleation, resulting in grain refinement of various phases within the solder bulk [10,11]. SEM-BSE micrographs (Figure 7) are used to show the microstructural differences between the reference SAC0307 and the ZrO_2_ composite joints. Figure 7a–c show the microstructure after sample preparation (0 h) (marked by a green line), and Figure 7d–f present the microstructure after 4000 h TH test (marked by a blue line). The solder matrix contains mostly β-Sn grains, Cu_6_Sn_5_ IMCs (darker gray dots), and Ag_3_Sn IMCs (white dots) [39,40]. Ag_3_Sn IMCs are located exclusively at the grain boundaries, serving as indicators of those boundaries. In Figure 7a–c, some of the presumed Sn grain boundaries are marked by red dashed lines.

While the average size of the Sn grains was 30–40 µm in the reference SAC0307 (Figure 7a), the average Sn grain size decreased after the addition of ZrO_2_ to 8–10 µm and 10–15 µm in the SAC-0.5ZrO_2_(np) and SAC-0.5ZrO_2_(nf) composite solder joints, respectively (Figure 7b,c). In the lower right corner of Figure 7e, the finer Sn grain structure is directly observable due to the OPS polishing of the cross-section. In the composite solder joints, the IMCs were more dispersed throughout the solder bulk compared to the reference joints (Figure 7a). The size of the “Ag_3_Sn islands” decreased considerably, particularly in the SAC-0.5ZrO_2_(np) (Figure 7b). These microstructural changes resulted in the mechanical improvement of the ZrO_2_ composite joints (Figure 1). Similar results were reported by Gain et al. [15,17]. The refinement of IMCs in the composite solder joints can be explained by the following: according to heterogeneous nucleation theory, the presence of ZrO_2_ NPs in the molten solder decreases the thermodynamic energy of IMCs nucleation since they prefer to nucleate on the ZrO_2_ NPs. The increased nucleation density results in more dispersed IMCs in the solder bulk [41]. Furthermore, according to the adsorption theory, the presence of ZrO_2_ NPs as a surface-active material decreases the surface energy of the IMC grains, which decreases their growth velocity, resulting in smaller IMC grains [28,41].

Long cracks formed between the Cu_6_Sn_5_ IMC layer and the solder bulk during the TH test. Such cracks were typical for all types of solder joint (Figure 7d–f). Figure 7f shows a highly magnified SEM micrograph of this phenomenon. Such cracks could have contributed to a significant drop in the shear forces during the TH test (Figure 1). Notably, in Figure 7d, corrosion spread extensively within the solder joint, with the corrosion depth reaching 50 µm. A more detailed EDS analysis of the corrosion spots can be seen later, in Figure 9.

### 4.2. DFT Calculations of the Sn Cluster on the ZrO_2_

**Figure 8:**
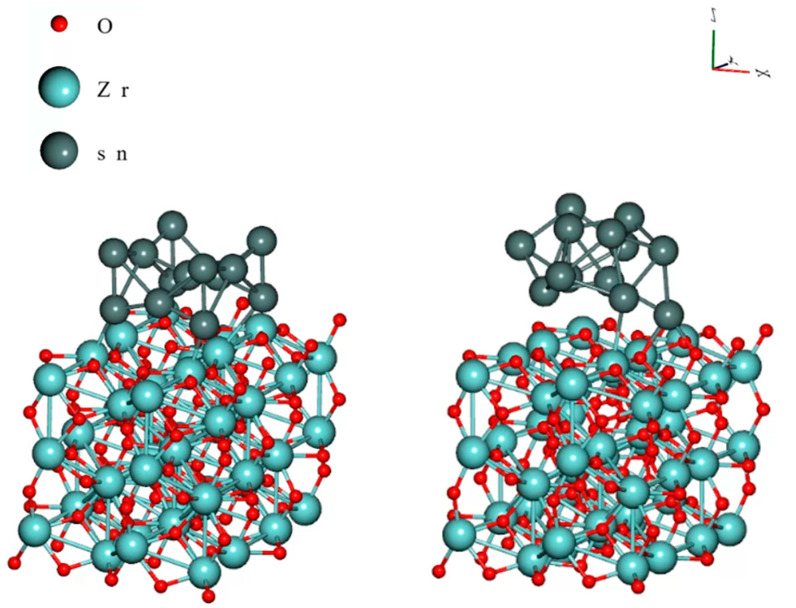
DFT calculation of the Sn cluster on the ZrO_2_ crystal, the initial position is left, and the optimized position is right.

We conducted DFT calculations in order to study the possible interaction between the ZrO_2_ NPs and the Sn atoms. We simulated the absorption energy of a Sn cluster on the ZrO_2_ monoclinic crystal (110) surface. The wave functions were expanded in plane waves up to a kinetic energy of 47.1 Ry, together with a cutoff at 424.0 Ry for the augmented density. In Figure 8, the left side shows the initial position of the calculation (an Sn cluster was over a ZrO_2_ crystal), and the right side presents the DFT results.

The binding energy (*E_B_*) between the ZrO_2_ and the Sn is as follows:

(1) $$E_{B}=\left(E_{tot}-E_{ZrO_{2}}-E_{Sn}\right)/n$$

where E*_tot_* is the total system energy, E*_ZrO2_* is the ZrO_2_ crystal energy, E*_sn_* is the Sn cluster energy, and n is the Sn atom number directly on the surface of the ZrO_2_ crystal (n = 4). DFT proved that Sn can bond on the ZrO_2_ surface with 1.15 eV binding energy. However, this binding energy is relatively low, which could result in instability in the system. In our previous study, we found much higher binding energy between Sn and TiO_2_ (2.12 eV), where increased corrosion resistance was observed [30]. In the present study, ZrO_2_ decreased the corrosion resistance of the composite solder joints, so it was worth examining the corrosion processes in our system.

### 4.3. Corrosion Process of the Composite Solder Joints

Under normal circumstances, Sn is stable in water; however, when it comes in contact with hot water vapour (like during our TH test) Sn(II) and Sn(IV) oxides form. However, this is only a thin Sn-oxide layer, which should not cause such large and deep corrosion spots, as presented in Figure 2, Figure 3, Figure 4 and Figure 5. In SAC solder alloys, the localized corrosion can act as well since the dispersed IMCs (Cu_6_Sn_5_, Cu_3_Sn, and Ag_3_Sn) in the solder bulk have higher standard reduction potential than Sn [42], which formed the anodic (the Sn) and the cathodic side (the IMCs) in our system. The condensed water film contained various contaminant ions (like Na, S, and Cl), so it could act as an electrolyte layer. This was probably the main reason for the corrosion to spread quickly into the solder bulk [43] (as demonstrated in Figure 2, Figure 3, Figure 4 and Figure 5). In this electrochemical cell, the Sn corroded and dissolved, Equations ((2) and (3)) [43], while the O_2_ depolarization was a parallel reaction on the cathode, Equation (4). The Sn^2+^/Sn^4+^ ions met with the OH- ions, they precipitated (KSP(Sn(OH)_4_) = 10^−57^ and KSP(Sn(OH)_2_) = 10^−27^) [44] and Sn(II)/Sn(IV) oxides formed according to Equations (5)–(8) [45]:

(2) $$Sn\to Sn^{2+}+2e^{-}$$

(3) $$Sn^{2+}\to Sn^{4+}+2e^{-}$$

(4) $$2H_{2}O+O_{2}+4e^{-}\to 4OH^{-}$$

(5) $$Sn^{2+}+2OH^{-}\to Sn{\left(OH\right)}_{2}$$

(6) $$Sn^{4+}+4OH^{-}\to Sn{\left(OH\right)}_{4}$$

(7) $$Sn{\left(OH\right)}_{2}\to SnO+H_{2}O$$

(8) $$Sn{\left(OH\right)}_{4}\to SnO_{2}+H_{2}O$$

**Figure 9:**
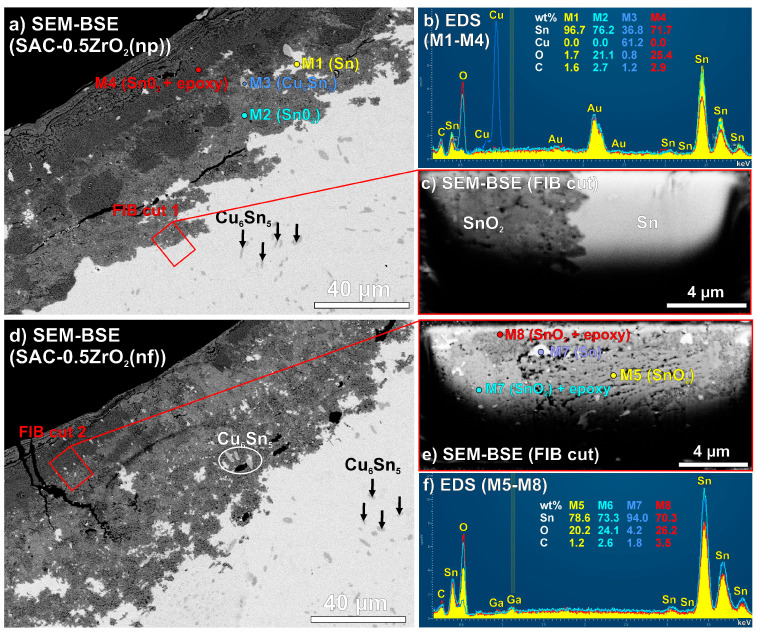
Investigation of localized corrosion in a SAC-ZrO_2_ solder joint: (**a**) SEM-BSE of SAC-0.5ZrO_2_(np); (**b**) EDS spectra (M1-M4); (**c**) SEM-BSE of FIB cut 1; (**d**) SEM-BSE of SAC-0.5ZrO_2_(nf); (**e**) SEM-BSE of FIB cut 2; (**f**) EDS spectra (M5–M8).

Further investigations were conducted on the corrosion spots to explore their composition and structure. Figure 9 presents a deeper analysis of corrosion spots in SAC-0.5ZrO_2_(np) and SAC-0.5ZrO_2_(nf) solder joints. The corrosion penetrated approximately 50–60 µm deep into the solder joints (Figure 9a,d). The SEM-BSE micrographs showed elemental differences in the corrosion spots. The upper parts of the figures were usually darker gray than the lower parts (Figure 9a,d). A series of EDS measurements proved that the upper part (darker gray) contained more oxygen (O) and carbon (C) than the lower part of the corrosion spots (lighter gray) (Figure 9a,b, M2 and M4). The lighter gray parts of the corrosion spots contained approximately 80 wt% Sn and 20 wt% O (Figure 9a,b, M2), which meant that it was primarily composed of SnO_2_. The formation of SnO_2_ has lower Gibbs free energy than SnO, so the formation of SnO_2_ was favorable. The observed increase of O and C at the upper parts of the corrosion spots could be caused by the sample preparation. The epoxy resin—used for the encapsulation of the solder joints—penetrated to upper Sn oxide, usually up to 10–20 µm depth. The M1 and M3 EDS measurements (Figure 9a,b) showed enclosed Sn and Cu_6_Sn_5_ by the corrosion spot.

A FIB cut was prepared (Figure 9c) at the boundary of a corrosion spot in the SAC-0.5ZrO_2_(np) solder joint (Figure 9a) to observe the structure of the SnO_2_ layer. The observation revealed that the SnO_2_ layer had a porous structure (containing plenty of holes), thus enhancing the corrosion propagation (according to Equations (2)–(8)) toward the solder bulk by bringing the condensed water into the solder joints. Another FIB cut was prepared (Figure 9e) at the boundary between the darker and lighter gray part of the corroded area of SAC-0.5ZrO_2_(nf) solder joint (Figure 9c), and further EDS analyses were performed on this cut. It was evident that the upper part of the corrosion spot (Figure 9e,f; M7 and M8) was more solid than the lower part (M5). This confirmed our assumptions about the filling of the porous SnO_2_ with epoxy resin during sample preparation. Enclosed Sn (M7) and likely Ag_3_Sn IMCs are also visible in Figure 9e.

**Figure 10:**
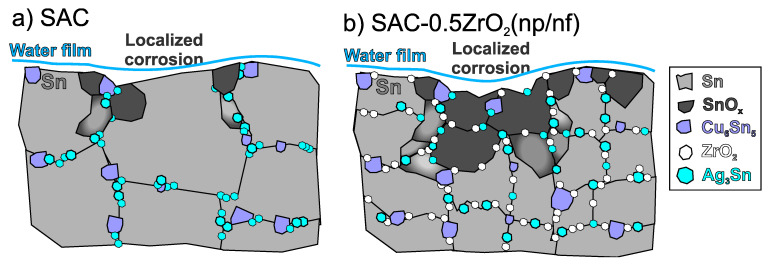
Corrosion of the solder joints: (**a**) simple corrosion in SAC solder joints; (**b**) enhanced corrosion in SAC-0.5ZrO_2_(np/nf) composite solder joints.

The corrosion processes discussed above (Equations (2)–(8)) occurred in the same manner across all sample types. The intensive corrosion of SAC-0.5ZrO_2_ solder joints could be explained by the refined microstructure of the composite solder joints. Figure 10 summarizes the corrosion differences between the reference SAC0307 and the composite solder joints. Generally, a refined grain structure is not favorable for corrosion resistance, as corrosion typically initiates at the grain boundaries, where the surface free energy is higher [46,47]. The refinement of the grain structure naturally led to an extensive grain boundary network in the composite solder joints resulting in a higher grain-boundary free energy for the system compared to the reference solder joints [48]. Another factor that could also facilitate corrosion was the increased dispersion of IMCs in the composite solder bulk, which might lead to the formation of more electrochemical cells than observed in the reference solder joints.

SAC-0.25ZrO_2_(np/nf) composite joints exhibited less pronounced microstructural refinement, which may explain their similar corrosion behavior to that of the reference SAC samples.

## 5. Conclusions

The mechanism and effects of different ZrO_2_ NPs incorporation into SAC0307 composite solder joints were investigated. Our main findings were the following:

- The application of ZrO_2_ NPs increased the initial shear force of the composite solder joints by 4–13.2%. During the 4000 h 85 °C/85% RH test, the shear force of the composite joints with 0.5 wt% ZrO_2_ NPs decreased by 29–35%, while the rest of the solder joints (reference and composite) lost only 12–12.5%.
- The composite solder joints with 0.5 wt% ZrO_2_ corroded twice more seriously than the rest of the samples. The intensive corrosion initiated the growth of numerous Sn whiskers. Interestingly, the correlation between the intensity of whiskering and the size of the corroded areas was weaker than initially expected. The ZrO_2_ composite solder joints produced a bit longer but not more whiskers than the reference SAC0307 joints.
- DFT simulations showed that Sn can bond to the ZrO_2_, but only with weak binding energy, which does not result in a stable system. ZrO_2_ nano-particles refined the microstructure of the solder joints, the β-Sn grain size was reduced, and the intermetallic compounds were more dispersed. This resulted in improved mechanical properties by dispersion strengthening but may have also reduced the corrosion resistance of the ZrO_2_ composite solder joints.
- The shape of the nano-particles did not have a major effect on the composite solder joints. While ZrO_2_ nano-particles improved the solder joint mechanical properties, their use is recommended only in non-corrosive environments, such as microelectronics for space applications. In corrosive climates, the weight fraction of ZrO_2_ NPs should not exceed 0.25 wt% to avoid reliability problems.

## Figures and Tables

**Table 1:** Tin whisker statistics.

Sample Name	Whisker Density [pcs./mm^2^]	Average Length [µm]	Maximum Length [µm]
Reference SAC	396	11.2 ± 4.3	69
SAC-0.25ZrO_2_(np)	441	11.9 ± 5.1	94
SAC-0.5ZrO_2_(np)	487	13.1 ± 5.7	118
SAC-0.25ZrO_2_(nf)	372	11.6 ± 4.9	82

## Data Availability

The raw/processed data required to reproduce these findings cannot be shared at this time as the data also form part of an ongoing study.
